# Higher triglyceride glucose-waist height ratio index is associated with higher prevalence of gallstone: a population-based study

**DOI:** 10.3389/fmed.2024.1481620

**Published:** 2024-09-27

**Authors:** Jianjun Wang, Sirui Chen, Xi Chen, Chuan Qin, Junchao Hu, Xintao Zeng, Hua Luo, Pei Yang, Huiwen Luo, Chuanpeng Yuan, Ruizi Shi, Decai Wang

**Affiliations:** ^1^Department of Hepatobiliary Surgery, Mianyang Central Hospital, School of Medicine, University of Electronic Science and Technology of China, Mianyang, China; ^2^NHC Key Laboratory of Nuclear Technology Medical Transformation, Mianyang Central Hospital, School of Medicine, University of Electronic Science and Technology of China, Mianyang, China; ^3^The Central Health Center of Longchi Town, Dujiangyan, China; ^4^Department of Urology, Mianyang Central Hospital, School of Medicine, University of Electronic Science and Technology of China, Mianyang, China

**Keywords:** gallstone, triglyceride glucose-waist height ratio, cross-sectional study, abdominal obesity, metabolic syndrome, National Health and Nutrition Examination Survey

## Abstract

**Background:**

The aim of this study is to evaluate the association between triglyceride glucose-waist height ratio (TyG-WHtR) index and the prevalence of gallstone disease (GSD), alongside the age at first gallstone surgery among adult populations within the United States.

**Methods:**

We screened participants using the National Health and Nutrition Examination Survey (NHANES). Logistic regression analysis, generalized additive modeling, smoothed curve fitting, and subgroup analysis were employed to assess the association between the TyG-WHtR index, prevalence of GSD, and the age at initial gallstone surgical intervention.

**Results:**

In this study, 3,728 participants were enrolled, among whom 395 individuals reported a prior history of GSD. The association between the TyG-WHtR index and the prevalence of GSD demonstrated a non-linear, positive association. After adjusting for all potential confounders, for each incremental unit rise in the TyG-WHtR index, there was a 47% escalation in the prevalence of GSD (OR = 1.47, 95% CI: 1.29, 1.68). Subgroup analyses indicated a more pronounced association between the TyG-WHtR index and the prevalence of GSD among individuals aged 20–80 years, females, non-Hispanic white population, non-Hispanic black population, other racial groups, and non-diabetic cohorts. Additionally, this study identified that the TyG-WHtR index may be negatively correlated with age at first surgical treatment of gallstones.

**Conclusion:**

An elevated TyG-WHtR index demonstrates a positive association with the prevalence of GSD. However, more prospective studies are needed to validate our findings.

## 1 Introduction

Gallstone disease (GSD) stands as a prevalent affliction within the global spectrum of digestive system disorders and constitutes a recognized risk element for gallbladder cancer (1). Epidemiological surveys have revealed that the incidence of GSD varies considerably between regions and ethnicities. For instance, the prevalence of GSD is higher in the United States and Europe than in Asia (2). Within the United States, the occurrence rate of GSD ranges from approximately 10–15% among Caucasians to as high as 70% among individuals of Indian descent (3). About 75% of patients with GSD have no obvious clinical symptoms during the initial phases of the condition and are often diagnosed during physical health examinations (4). Typical clinical symptoms of GSD include epigastric pain, nausea, vomiting, and anorexia triggered by fatty foods. A small number of patients develop acute cholangitis, acute pancreatitis, or infectious shock due to stones entering the common bile duct (5).

Although significant progress has been made in the early detection and management, the precise etiology of GSD remains incompletely understood. Factors such as age, sex, nutrition, environment, genetics, cholesterol metabolism disorders, gallbladder dysfunction, and gut microorganisms are involved in the pathogenesis of GSD (4, 6, 7). Notably, studies have increasingly highlighted the role of metabolic disorders and obesity in the pathogenesis of GSD (8–10). Within this array of factors, insulin resistance (IR) has surfaced as a pivotal element in the complex interplay between metabolic dysregulation and associated pathologies. The triglyceride-glucose (TyG) index serves as a reliable surrogate indicator of IR and has been validated as a useful tool for evaluating conditions such as diabetes mellitus, non-alcoholic fatty liver disease, and cardiovascular disease (11–13). However, the TyG index fails to account for changes in adipose tissue distribution, especially in central obesity, which is strongly associated with metabolic abnormalities. The waist-to-height ratio (WHtR) is widely used as a simple visual measure to evaluate adipose tissue distribution and central obesity (14). By integrating the TyG index and the WHtR, the TyG-WHtR index can provide a more comprehensive assessment of IR, metabolic disorders, and adipose tissue distribution. Xuan et al. demonstrated that the TyG-WHtR index outperforms the TyG index and other markers in identifying individuals at risk for type 2 diabetes mellitus (15).

However, despite evidence that metabolic disorders and obesity are inextricably linked to the development of GSD, reliable indicators for predicting and assessing the risk of GSD are still lacking. Therefore, the aim of this study was to assess the significance of the TyG-WHtR index in the development of GSD.

## 2 Methods

### 2.1 Source of data and study population

**Figure 1:**
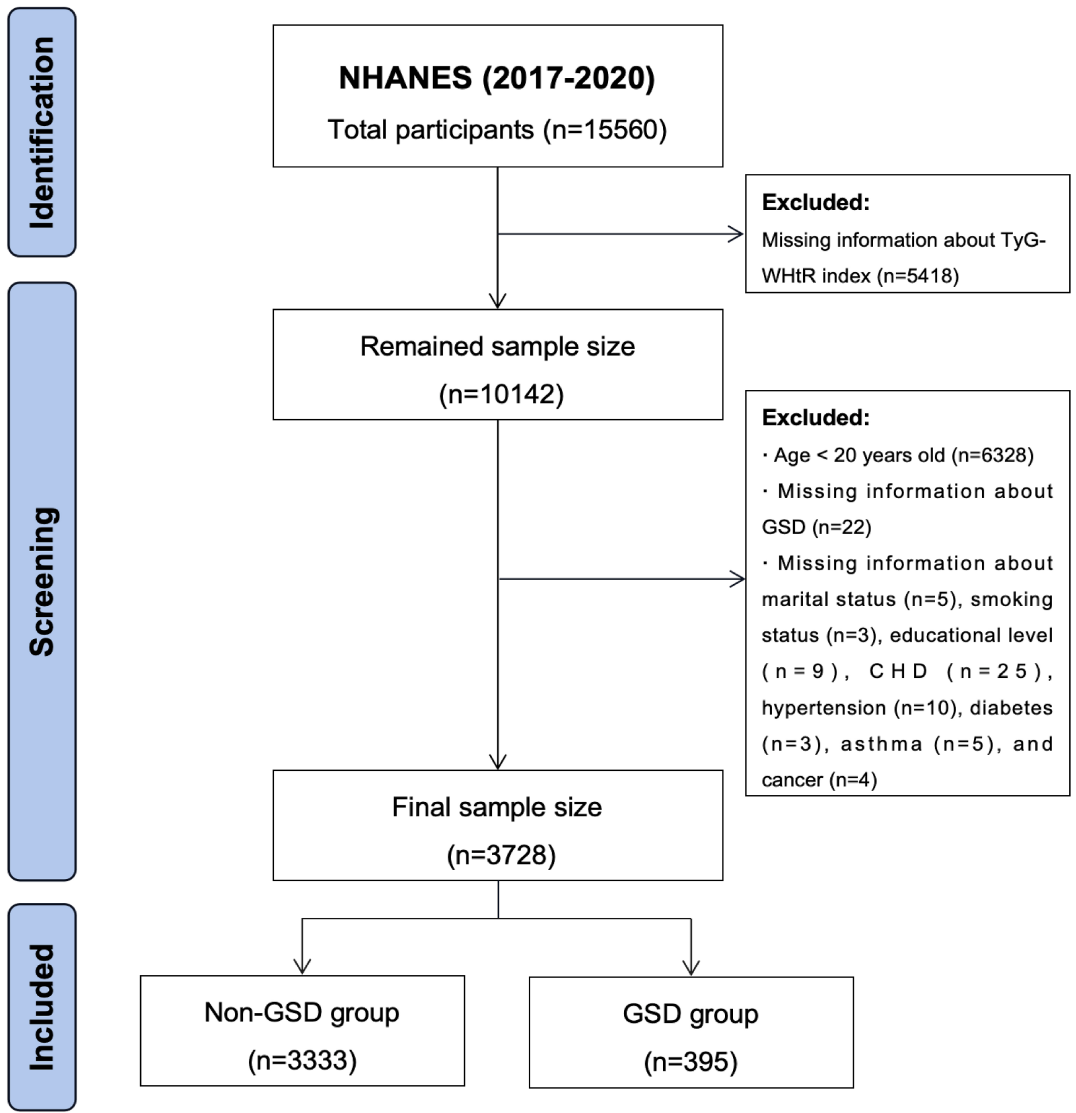
Flowchart for participants from NHANES (2017–2020).

All data utilized in this study were obtained from the National Health and Nutrition Examination Survey (NHANES), an open-source database officially maintained by the Centers for Disease Control and Prevention (CDC). The NHANES undergoes biennial updates and typically encompasses a population of around 10,000 individuals per cycle. Due to the prevalence of COVID-19, the NHANES program was suspended in March 2020. Information on GSD was available only from 2017 to March 2020, so data from this period were used for the study. Since the questionnaires on GSD were only available for adults aged 20 years and older, participants younger than 20 years were excluded from the study. The detailed exclusion criteria were established based on the study objectives (Figure 1). Ultimately, 3,728 individuals were included in this study, of whom 395 had a history of GSD.

### 2.2 Data collection and parameter definition

Questionnaires were employed to ascertain the presence of GSD among participants and to document their age at the initial gallbladder surgery for GSD. The covariates included in this study comprised five main components: demographic variables, comorbidities, dietary intake factors, laboratory results, and others (Table 1). TyG-WHtR index served as the independent variable of interest. The TyG index = Ln (fasting triglycerides [mg/dL] × fasting glucose [mg/dL]/2) (16). The WHtR was defined as waist circumference (WC) divided by height (17). The TyG-WHtR index was computed using the formula: TyG-WHtR index = TyG index × WHtR (18).

**Table 1:** Covariates extracted from NHANES.

Items	Composition
Demographic variables	Age, gender, race
Comorbidities	Hypertension, diabetes, asthma, CHD, cancer
Laboratory results	Crea, TG, TC, HDL-C, LDL-C, ALT, AST, HbA1c, Ferritin
Dietary intake factors	Total sugar, total kcal, total fat, total water
Others	BMI, physical activity, marital status, alcohol consumption, education level, smoking status, PIR

NHANES, the National Health and Nutrition Examination Survey; CHD, coronary heart disease; Crea, serum creatinine; TG, triglyceride; TC, total cholesterol; HDL-C, high density lipoprotein cholesterol; LDL-C, low density lipoprotein cholesterol; ALT, alanine aminotransferase; AST, aspartate aminotransferase; HbA1c, glycosylated hemoglobin; BMI, body mass index; PIR, poverty income ratio.

All subjects enrolled in this study underwent a 24-h dietary recall process; thus, the mean intake derived from the two recalls was utilized for the analytical procedures. Detailed measurement procedures for all variables are available on the official NHANES website at www.cdc.gov/nchs/nhanes/. All procedures in compliance with the U.S. Department of Health and Human Services (HHS) Policy for the Protection of Human Research Subjects were followed throughout the NHANES protocols. These protocols are subject to annual review and standardization by the NCHS Research Ethics Review Board. All individuals participating in the NHANES study provided written informed consent; therefore, no further permissions or ethical reviews were necessary.

### 2.3 Outcome indicators

In this study, the prevalence of GSD and the age of patients at the time of their initial gallbladder surgery for GSD were considered as the outcome variables.

### 2.4 Statistical analysis

For normally distributed data, continuous variables were presented as mean ± standard deviation (SD), while for skewed data, continuous variables were described as median (interquartile range [IQR]). Categorical variables were represented as counts and percentages. Comparisons of continuous variables were conducted using t-tests or one-way ANOVA, whereas Pearson’s χ2 test and Fisher’s exact test were employed for comparisons of categorical variables. In accordance with established protocols, multivariate logistic regression models were employed to examine the association among the TyG-WHtR index, various TyG-WHtR tertiles, and the prevalence of GSD across diverse models. Model 1 served as the baseline model without any covariate adjustments. Model 2 was adjusted for age, sex, race, and marital status. Model 3 included adjustments for all potential confounders. The relationship between the TyG-WHtR index and the prevalence of GSD, as well as the age at initial gallstone surgery, was additionally assessed through generalized additive model (GAM) regression and smoothed curve fitting employing the penalized spline method. Upon identification of a non-linear relationship, a natural spline test was utilized to determine the values of inflection points. Subgroup analyses were conducted considering age, sex, race, body mass index (BMI), hypertension, and diabetes. Statistical significance was defined as *p* < 0.05 in this study. All analyses were performed using R version 4.0.2 (http://www.R-project.org; R Foundation for Statistical Computing) and Empower (www.empowerstats.com; X&Y Solutions Inc., Boston, MA, United States).

## 3 Results

### 3.1 Baseline characteristics of the study population

A total of 3,728 individuals were enrolled in this study, among whom 395 were identified as individuals with GSD based on self-reporting. Table 2 presents the baseline demographics of the enrolled participants in the study. The TyG-WHtR index was 5.1 (4.4–5.9) in non-GSD participants and 5.7 (5.1–6.5) in participants with GSD, and the disparity between the two cohorts was found to be statistically significant ( *p* < 0.001). Moreover, notable distinctions were observed between the two cohorts concerning age, sex, race, comorbidities, serum creatinine, triglycerides, glycosylated hemoglobin, BMI, physical activity, marital status, alcohol consumption, and smoking status (all *p* < 0.05).

**Table 2:** Baseline characteristics of participants.

Variable	Non-GSD group (*n* = 3,333)	GSD group (*n* = 395)	*p*-value
TyG-WHtR index	5.1 (4.4–5.9)	5.7 (5.1–6.5)	<0.001
Demographic variables			
Age (years)	51 (35–63)	59 (45–70)	<0.001
Gender			<0.001
Female	1,637 (49.11)	283 (71.65)	
Male	1,696 (50.89)	112 (28.35)	
Race (%)			<0.001
Non-Hispanic White population	1,108 (33.24)	156 (39.49)	
Non-Hispanic Black population	874 (26.22)	68 (17.22)	
Mexican American	418 (12.54)	58 (14.68)	
Other race	933 (27.99)	113 (28.61)	
Comorbidities			
Hypertension (%)			<0.001
No	2,110 (63.31)	180 (45.57)	
Yes	1,218 (36.54)	215 (54.43)	
Unclear	5 (0.15)	0 (0.00)	
Diabetes (%)			<0.001
No	2,741 (82.24)	278 (70.38)	
Yes	487 (14.61)	106 (26.84)	
Borderline	103 (3.09)	11 (2.78)	
Unclear	2 (0.06)	0 (0.00)	
Asthma (%)			<0.001
No	2,822 (84.67)	302 (76.46)	
Yes	508 (15.24)	93 (23.54)	
Unclear	3 (0.09)	0 (0.00)	
Coronary heart disease (%)			<0.001
No	3,193 (95.80)	362 (91.65)	
Yes	126 (3.78)	31 (7.85)	
Unclear	14 (0.42)	2 (0.50)	
Cancer (%)			<0.001
No	3,014 (90.43)	323 (81.77)	
Yes	317 (9.51)	71 (17.97)	
Unclear	2 (0.06)	1 (0.25)	
Laboratory results			
Crea (mg/dl)	74.26 (61.88–88.40)	70.72 (59.23–84.86)	0.01
TG (mmol/L)	1.15 (0.82–1.66)	1.36 (0.97–1.83)	<0.001
TC (mmol/L)	4.68 (4.03–5.41)	4.55 (3.96–5.38)	0.18
HDL-C (mmol/L)	1.32 (1.09–1.60)	1.29 (1.09–1.58)	0.23
LDL-C (mmol/L)	2.79 (2.22–3.41)	2.69 (2.12–3.44)	0.10
ALT (U/L)	17 (13–25)	18 (13–26)	0.87
AST (U/L)	19 (16–24)	18 (15–23)	0.10
HbA1c (%)	5.6 (5.3–6.0)	5.7 (5.4–6.3)	<0.001
Ferritin (ng/ml)	110.0 (53.8–205.0)	97.0 (46.6–179.0)	0.02
Dietary intake factors			
Total sugar	88.63 (54.21–137.06)	84.36 (54.23–126.67)	0.30
Total kcal	1978 (1443–2,667)	1773 (1296–2,412)	<0.001
Total fat	78.05 (52.79–111.97)	72.69 (49.46–103.29)	0.01
Total water	1920 (720–3,480)	1710 (630–3,600)	0.51
Others			
BMI	28.3 (24.5–33.1)	31.7 (27.5–37.5)	<0.001
Physical activity (%)			0.004
Never	982 (29.46)	142 (35.95)	
Moderate	1,117 (33.51)	138 (34.94)	
Vigorous	1,234 (37.02)	115 (29.11)	
Marital status (%)			<0.001
Cohabitation	1971 (59.14)	234 (59.24)	
Solitude	702 (21.06)	109 (27.59)	
Never married	660 (19.80)	52 (13.16)	
Alcohol consumption (%)			<0.001
Never	280 (8.40)	29 (7.342)	
Ever	613 (18.39)	117 (29.62)	
Now	2,294 (68.83)	227 (57.47)	
Unclear	146 (4.38)	22 (5.57)	
Education level			0.67
Less than high school	628 (18.84)	79 (20.00)	
High school	787 (23.61)	98 (24.81)	
More than high school	1918 (57.55)	218 (55.19)	
Smoking status (%)			0.003
Never	1910 (57.31)	216 (54.68)	
Ever	781 (23.43)	121 (30.63)	
Now	642 (19.26)	58 (14.68)	
PIR			0.06
< 1.3	781 (23.43)	88 (22.28)	
≥ 1.3–<3.5	1,133 (33.99)	161 (40.76)	
≥ 3.5	968 (29.04)	99 (25.06)	
Unclear	451 (13.53)	47 (11.90)	

### 3.2 Logistic regression results between the TyG-WHtR index and GSD

The logistic regression analysis unveiled a direct relationship between the TyG-WHtR index and the prevalence of GSD (Table 3). This positive association persisted in the fully adjusted model (Model 3) (OR = 1.47, 95% CI: 1.29, 1.68). These results suggest that for every 1-unit increase in the TyG-WHtR index, the prevalence of GSD increases by 47%. Furthermore, when the TyG-WHtR index was converted from a continuous variable to a categorical variable (tertiles) for sensitivity analysis, there was a 1.06-fold rise in the prevalence of GSD in Tertile 3 (OR = 2.06, 95% CI: 1.41, 2.99) compared to Tertile 1, the lowest TyG-WHtR index tertile.

**Table 3:** Logistic regression analysis between TyG-WHtR index with GSD prevalence.

Characteristic	Model 1 OR (95%CI)	Model 2 OR (95%CI)	Model 3 OR (95%CI)
TyG-WHtR index	1.68 (1.54–1.85)	1.55 (1.40–1.71)	1.47 (1.29–1.68)
Categories			
Tertile 1	1	1	1
Tertile 2	2.18 (1.60–2.99)	1.77 (1.28–2.45)	1.55 (1.10–2.19)
Tertile 3	3.76 (2.80–5.05)	2.78 (2.04–3.78)	2.06 (1.41–2.99)

Model 1 = no covariates were adjusted. Model 2 = Model 1 + age, gender, race, and marital status were adjusted. Model 3 = Model 2 + education, physical activity, smoking status, alcohol consumption, diabetes, hypertension, asthma, cancers, CHD, Crea, TC, TG, HDL-C, LDL-C, ALT, AST, HbA1c, ferritin, PIR, total kcal, total fat, and total sugar were adjusted.

### 3.3 Dose–response and threshold effects of the TyG-WHtR index on GSD prevalence

**Figure 2:**
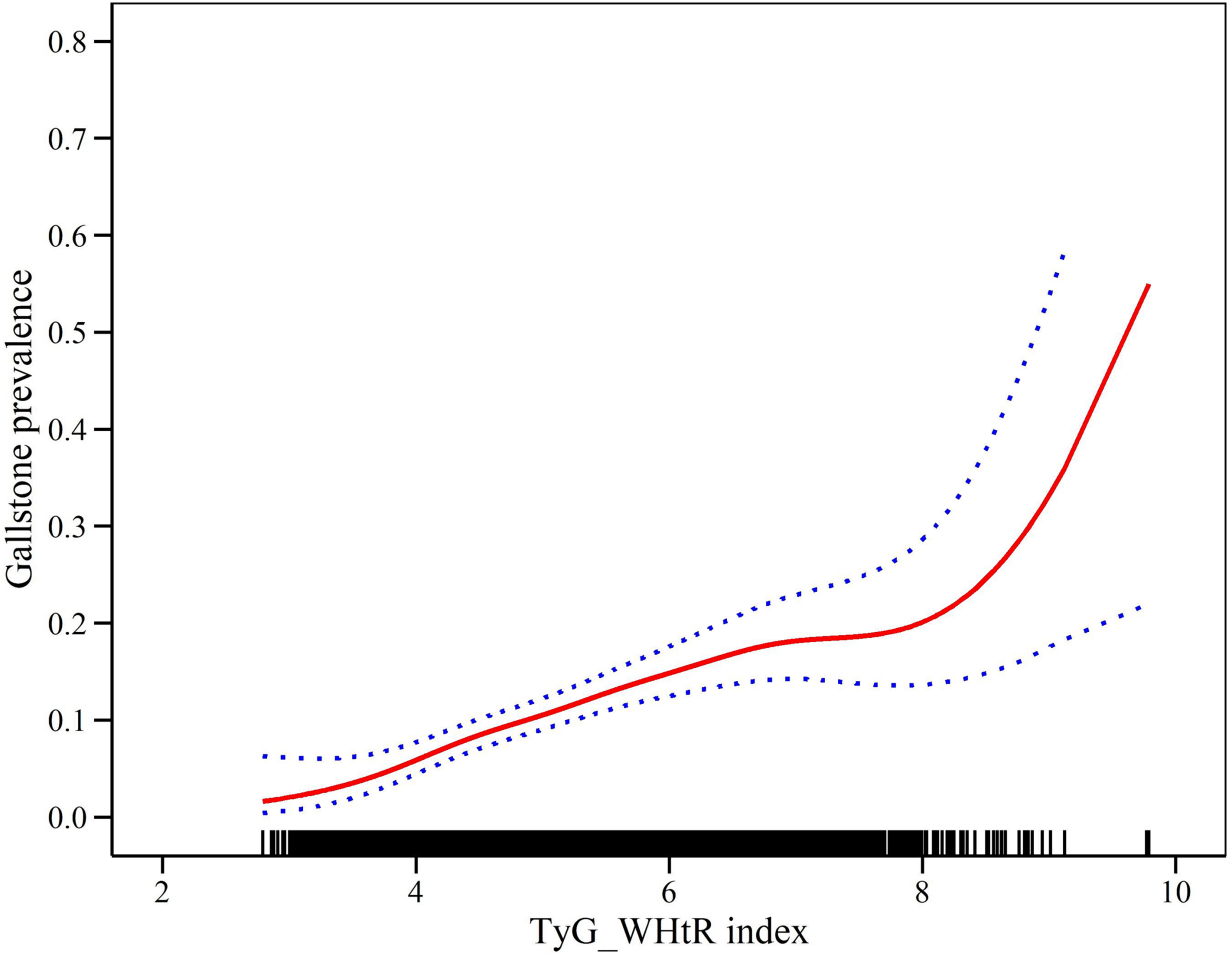
Density dose–response relationship between TyG-WHtR index and the prevalence of GSD. The area between the upper and lower dashed lines represents the 95% CI.

Generalized additive modeling and smooth curve fitting were employed to further investigate the association between the TyG-WHtR index and the prevalence of GSD. Figure 2 illustrates the non-linear association (positive) between the TyG-WHtR index and the prevalence of GSD. Taking into account the saturation threshold effect, the likelihood ratio test indicated that the optimal threshold for the TyG-WHtR was 4.15 (Table 4).

**Table 4:** Two-piecewise linear regression and logarithmic likelihood ratio test explained the threshold effect analysis of TyG-WHtR index with GSD prevalence.

TyG-WHtR index	ULR test	PLR test	LRT test
	OR (95%CI)	OR (95%CI)	p-value
< 4.15	1.15 (1.27–1.65)	11.66 (2.10–64.70)	0.005
> = 4.15		1.36 (1.19–1.56)	

ULR, univariate linear regression; PLR, piecewise linear regression; LRT, logarithmic likelihood ratio test, statistically significant: p < 0.05.

### 3.4 Subgroup analysis

To reinforce the validity of the findings, a subgroup analysis was undertaken. When adjusted for all potential confounders, we found that the association between TyG-WHtR index and GSD prevalence was more significant in these populations: Age group 20–39 years (OR = 1.80, 95% CI: 1.32, 2.44); Age group 40–59 years (OR = 1. 36, 95% CI: 1.09, 1.71); Age group 60–80 years (OR = 1.41, 95% CI: 1.13, 1.76); Female group (OR = 1.56, 95% CI: 1.33, 1.82); Non-Hispanic white population (OR = 1.48, 95% CI: 1.17, 1.85); Non-Hispanic black population (OR = 1.46, 95% CI: 1.08, 1.96); Other race group (OR = 1.55, 95% CI: 1.18, 2.04); Non-hypertensive population group (OR = 1.63, 95% CI: 1.33, 1.99); Hypertensive population group (OR = 1.32, 95% CI: 1.10, 1.59); Non-diabetic population group (OR = 1.57, 95% CI: 1.34, 1.84). More details of the results are presented in Table 5.

**Table 5:** Subgroup regression analysis between TyG-WHtR index with GSD prevalence.

Characteristic	Model 1 OR (95%CI)	Model 2 OR (95%CI)	Model 3 OR (95%CI)
Stratified by age (years)			
20–39	2.10 (1.72–2.58)	1.92 (1.54–2.40)	1.80 (1.32–2.44)
40–59	1.45 (1.24–1.70)	1.41 (1.20–1.66)	1.36 (1.09–1.71)
60–80	1.56 (1.33–1.81)	1.47 (1.25–1.73)	1.41 (1.13–1.76)
Stratified by gender			
Female	1.67 (1.50–1.86)	1.62 (1.44–1.81)	1.56 (1.33–1.82)
Male	1.50 (1.26–1.79)	1.36 (1.12–1.66)	1.29 (0.98–1.69)
Stratified by race			
Non-Hispanic White population	1.71 (1.47–1.98)	1.60 (1.37–1.86)	1.48 (1.17–1.85)
Non-Hispanic Black population	1.72 (1.40–2.12)	1.56 (1.25–1.95)	1.46 (1.08–1.96)
Mexican American	1.46 (1.13–1.90)	1.14 (0.85–1.53)	1.28 (0.86–1.92)
Other Race	1.72 (1.43–2.06)	1.66 (1.37–2.01)	1.55 (1.18–2.04)
Stratified by BMI			
≤ 24.9	2.30 (1.46–3.64)	1.20 (0.67–2.13)	1.10 (0.46–2.64)
25–29.9	1.73 (1.25–2.38)	1.11 (0.75–1.64)	1.12 (0.60–2.07)
≥ 30	1.55 (1.34–1.80)	1.34 (1.14–1.57)	1.22 (0.99–1.50)
Stratified by hypertension			
No	1.69 (1.47–1.94)	1.55 (1.33–1.79)	1.63 (1.33–1.99)
Yes	1.52 (1.33–1.74)	1.44 (1.25–1.66)	1.32 (1.10–1.59)
Stratified by diabetes			
No	1.72 (1.53–1.93)	1.57 (1.39–1.78)	1.57 (1.34–1.84)
Yes	1.36 (1.12–1.66)	1.33 (1.08–1.65)	1.20 (0.90–1.60)

### 3.5 Density dose–response relationship between TyG-WHtR index and age at first gallstone surgery

**Figure 3:**
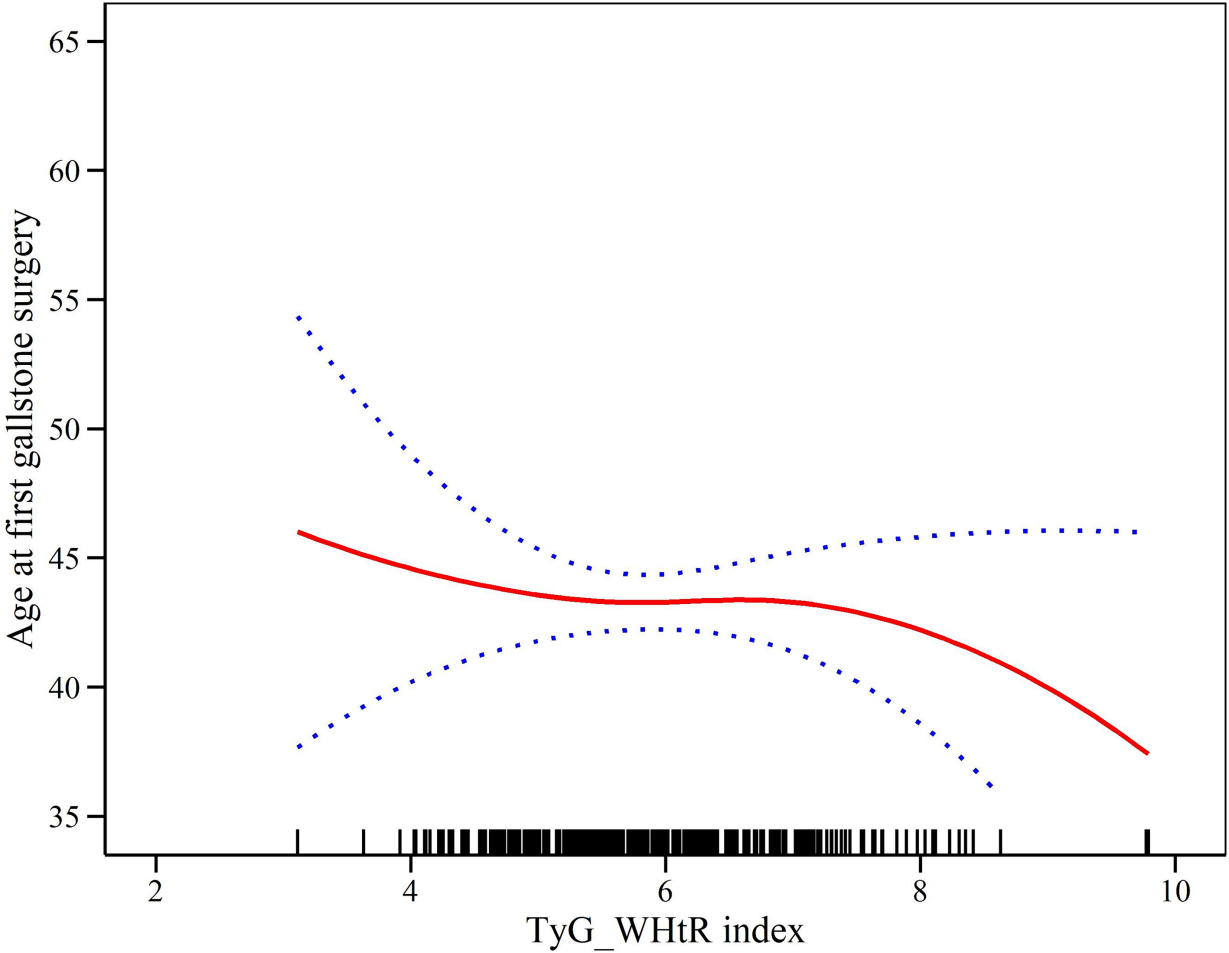
Density dose–response relationship between TyG-WHtR index and age at first gallstone surgery. The area between the upper and lower dashed lines represents the 95% CI.

In order to delve deeper into the association between the TyG-WHtR index and the age of initial gallstone surgery, we employed generalized additive modeling along with smoothed curve fitting techniques. Figure 3 shows that the TyG-WHtR index may be negatively correlated with age at first gallstone surgery.

## 4 Discussion

This study represents the inaugural comprehensive analysis exploring the interplay among the TyG-WHtR index, the incidence of GSD, and the age of initial gallstone surgical intervention. We observed a non-linear positive association between the TyG-WHtR index and the incidence of GSD, suggesting that for each 1-unit rise in the TyG-WHtR index, the prevalence of GSD escalated by 47%. Moreover, upon categorizing the TyG-WHtR index from a continuous to a categorical format (tertiles), we observed that individuals in the highest TyG-WHtR index tertile exhibited a 1.06-fold higher likelihood of GSD incidence compared to those in the lowest tertile. Subgroup analyses indicated that the association between the TyG-WHtR index and the prevalence of GSD exhibited greater significance among individuals aged 20–80 years, females, non-Hispanic white population, non-Hispanic black population, other racial groups, and non-diabetic populations. Additionally, we found that an increased TyG-WHtR index was associated with an earlier age at first gallstone surgery.

The elevated prevalence of GSD presents a substantial public health concern on a global scale, with 1.5 million individuals in the United States alone seeking medical assistance for GSD in 2015 (19). As mentioned in the introduction, GSD pathogenesis is influenced by various factors. The incidence of GSD differs by sex, with females generally exhibiting a higher prevalence than males (20). This disparity is primarily attributed to hormone levels, particularly the biological effects of estrogen, which plays a pivotal role (21, 22). Estrogen can increase cholesterol deposition in bile, thereby promoting gallstone crystallization (22). Additionally, estrogen can affect gallbladder contractility, leading to prolonged bile retention, thereby increasing the risk of stone formation (21). Moreover, estrogen can alter the composition of bile constituents, further increasing the risk of stone development (21). Our results also confirm a higher prevalence of GSD in females compared to males. Most existing studies identify advanced age as a contributing risk factor for the onset of GSD (23). Nonetheless, a growing body of literature indicates ongoing debate regarding the classification of age as a significant risk factor for GSD. According to a recent study, influences such as obesity and disorders in cholesterol metabolism exert a more pronounced influence on the prevalence of GSD, particularly among younger populations (24). Genetic factors also influence GSD pathogenesis, with Caucasians generally exhibiting a higher prevalence of GSD compared to non-Hispanic black population (3). For example, in the United States, the prevalence among non-Hispanic white women is 16.6% compared to 8.6% in men, and among non-Hispanic black women, it is 13.9% compared to 5.3% in men (9). These differences may be attributed to a wide range of factors such as genetics, nutrition, lifestyle, and environment among different ethnic groups. For instance, Caucasians have greater access to higher-quality diets compared to most non-Hispanic black population, which may contribute to their higher GSD incidence, along with higher calorie intake and lifestyles (25, 26).

The contribution of metabolic abnormalities (such as IR, diabetes, and obesity) to the development of GSD is increasingly recognized in medical research. Studies have demonstrated a strong association between metabolic syndrome and the incidence of GSD (5, 27, 28). Obesity, which constitutes a core aspect of metabolic disorders, emerges as notably impactful in predisposing individuals to GSD. Several studies have revealed a significant association between obesity and GSD (10, 29). Obesity promotes stone formation mainly through several mechanisms. Firstly, obese patients are usually associated with abnormal cholesterol metabolism, and when cholesterol levels in the bile are too high, cholesterol precipitates and forms stones (8, 9, 30). Secondly, obese patients are often associated with metabolic syndrome and IR, which can also lead to weakened gallbladder motility, bile siltation in the gallbladder to form cholesterol crystals, and ultimately the formation of gallstones (30–32). In addition, obese patients are usually accompanied by hypertriglyceridemia, high levels of triglycerides will further affect cholesterol metabolism, exacerbate the oversaturation of bile, and increase the risk of gallstone formation (5, 8, 9, 31). Moreover, obese people often tend to consume high-calorie, low-fibre food, this diet structure will not only increase the intake of cholesterol, but also affect the normal contraction and emptying function of the gallbladder, which further promotes the formation of gallstones (10, 20, 22, 33). Numerous recent epidemiological studies have additionally proposed that individuals with metabolic conditions such as diabetes mellitus face a notably heightened susceptibility to GSD development (31, 33). Effective early management of blood glucose levels, including regular metformin administration, can contribute to reducing the development of GSD in patients with diabetes (34). Butyrylcholinesterase (BChE) is a non-specific cholinesterase that is associated with abnormal liver function (35, 36). Notably, elevated BChE activity may reflect the extent of lipid metabolism disorders and IR in the body, which may also exacerbate the risk of cholesterol stones (35, 36). In addition, BChE activity may be associated with systemic inflammation and oxidative stress, which also play a role in gallstone development (35, 36). However, the detailed relationship between BChE and GSD deserves further study.

IR is one of the core mechanisms by which metabolic disorders and obesity affect the prevalence of GSD. The TyG index has demonstrated its validity as a suitable substitute for IR (11–13). However, the TyG index does not account for variations in adipose tissue distribution. While WC can reflect central obesity, it does not clearly differentiate between subcutaneous and visceral adipose tissues. Research indicates that visceral adipose tissue contributes more significantly to IR development than subcutaneous adipose tissue (37). Previous research has emphasized that height is a crucial factor in adipose tissue distribution, making the WHtR a superior predictor compared to WC alone (14, 38). For instance, Paajanen et al. found that patients with short stature are more likely to develop coronary heart disease (39). Therefore, combining WC with height enhances the accuracy of assessing central obesity and metabolism. The TyG-WHtR index represents a straightforward and intuitive measure incorporating fasting plasma glucose, triglyceride levels, WC, and height (40). With the integration of the TyG index and WHtR, the TyG-WHtR index can provide a more comprehensive assessment of IR and adipose tissue distribution. Our findings further corroborated the strong association between the TyG-WHtR index and both the prevalence of GSD and the age at initial gallstone surgery.

To our knowledge, this study represents the initial study into the association between the TyG-WHtR index and the prevalence of GSD. In summary, our study identified a non-linear, positive association between the TyG-WHtR index and the prevalence of GSD, demonstrating a 47% escalation in incidence for every 1-unit increment in the TyG-WHtR index. Our study possesses several strengths. First, this study represents the pioneering effort to delineate the influence of the TyG-WHtR index on the prevalence of GSD. Second, the NHANES participants constituted a representative sample from the United States, adhering closely to a meticulously designed study protocol and undergoing rigorous quality control measures, thus ensuring the reliability of our conclusions. Third, the NHANES offered comprehensive demographic and metabolic information, along with subsequent follow-up assessments, facilitating the adjustment for potential confounding variables in our multivariate analyses. Last, we conducted subgroup analyses to ensure the generalizability of our results across different populations, underscoring the thoroughness and robustness of our study. TyG-WHtR index may have many potential applications in the future. For example, the positive association between the TyG-WHtR index and the prevalence of GSD suggests that it could be used as a valuable biomarker for predicting an individual’s risk of developing GSD, particularly in women, non-diabetics, and ethnically diverse groups. By monitoring this index, clinicians can identify high-risk populations and thus develop early intervention and prevention measures. In addition, the TyG-WHtR index is strongly associated with metabolic syndrome and could be used in the future as part of the criteria in health screening, especially to identify individuals with metabolic abnormalities associated with GSD. In public health policy, the TyG-WHtR index may be used to guide prevention strategies for GSD, especially in obese, metabolically abnormal, and high-risk populations.

However, our study also has some limitations that should be acknowledged. First, given its cross-sectional design, we cannot establish a causal relationship between the TyG-WHtR index and the prevalence of GSD. Second, the diagnosis of GSD relied on a questionnaire, potentially introducing recall (recollection) bias. Third, due to the design of the NHANES, we could only measure fasting glucose and triglyceride levels at a single time point during baseline assessment; however, these laboratory parameters may change during follow-up. Last, despite adjusting for numerous possible confounders, we could not completely eliminate the effects of other unmeasured confounders.

Despite these limitations, this study is clinically important. The TyG-WHtR index represents a novel biomarker that integrates both IR and adipose tissue distribution, offering a more comprehensive assessment of metabolic health. Our study also revealed, for the first time, an association between the TyG-WHtR index and the prevalence of GSD, offering compelling evidence for the TyG-WHtR index’s predictive value in GSD development. In the future, conducting a multicenter prospective cohort study will be essential to explore the capacity of the TyG-WHtR index as an independent predictor of GSD incidence.

## 5 Conclusion

A higher TyG-WHtR index is positively associated with the prevalence of GSD and may be related to an earlier age at first gallstone surgery. A multicenter prospective cohort study is needed to investigate the potential of the TyG-WHtR index as an independent predictor of GSD prevalence.

## Data Availability

The original contributions presented in the study are included in the article/supplementary material, further inquiries can be directed to the corresponding authors.
